# When AHR signaling pathways meet viral infections

**DOI:** 10.1186/s12964-023-01058-8

**Published:** 2023-02-24

**Authors:** Jieke Hu, Yuan Ding, Wen Liu, Shuzhen Liu

**Affiliations:** 1 Department of Blood Transfusion, The Affiliated Hospital of Qingdao University, No. 1677 Wutaishan Road, Qingdao, 266555 China; 2 Department of Pathogenic Biology, Qingdao University Medical College, 308 Ningxia Road, Qingdao, 266071 China; 3 Department of Special Examination, Qingdao Women & Children Hospital, Qingdao, 266035 China

**Keywords:** Aryl hydrocarbon receptor, Virus infections, Signaling pathway

## Abstract

Aryl hydrocarbon receptor (AHR) is a ligand-dependent transcriptional factor widely expressed among immune, epithelial, endothelial and stromal cells in barrier tissues. It can be activated by small molecules provided by pollutants, microorganisms, food, and metabolism. It has been demonstrated that AHR plays an important role in modulating the response to many microbial pathogens, and the abnormal expression of AHR signaling pathways may disrupt endocrine, cause immunotoxicity, and even lead to the occurrence of cancer. Most humans are infected with at least one known human cancer virus. While the initial infection with these viruses does not cause major disease, the metabolic activity of infected cells changes, thus affecting the activation of oncogenic signaling pathways. In the past few years, lots of studies have shown that viral infections can affect disease progression by regulating the transmission of multiple signaling pathways. This review aims to discuss the potential effects of virus infections on AHR signaling pathways so that we may find a new strategy to minimize the adverse effects of the AHR pathway on diseases.

**Video Abstract**

**Supplementary Information:**

The online version contains supplementary material available at 10.1186/s12964-023-01058-8.

## Background

Diseases caused by virus infections account for millions of lost disability-adjusted life years (DALYs, a measure of disease burden) [1]. Increasing evidence suggests that the environment is an important factor affecting the host's response to virus infections. However, how environmental factors play a role in regulating the host's response to virus infections remains to be explored [2]. As an important environment-sensing ligand-inducible transcription factor, AHR plays a non-negligible role after virus infections. In addition, AHR can affect tumor growth, survival, migration and invasion by participating in cell proliferation, apoptosis and immune metabolism process [3]. In particular, the function of AHR has two sides: under the stimulation of polycyclic aromatic hydrocarbons (PAHs), halogenated aromatic hydrocarbons (HAHs) and other ligands, AHR can promote tumorigenesis and facilitate virus replication in vivo [4]. However, after activation by benzothiazoles, aminoflavone (AF), and other compounds, it can function as a tumor suppressor [5]. The powerful biological function of AHR has attracted researchers to explore it continuously, hoping to use AHR as a breakthrough to provide the basis for the treatment of related diseases. At present, AHR-targeting drugs are mainly AHR agonists, selective AHR modulators (SahRMs) and AHR antagonists, they can alter AHR activity in a ligand-dependent manner and affect the transmission of related signaling pathways [6].

Considering that AHR does not only function as a receptor, it is also a special E3 ubiquitin ligase and a transcription factor located in nucleus after being stimulated, the mechanism of its action is complex and variable [7, 8]. At the same time, the situation of the virus infection is even more changeable. Some are latent infection, while others are rapidly lytic replication. At different stages of virus infection, the products encoded by the virus have intricate effects on the host, and the immune response against the virus is also specific [9]. That is to say, it is still essential for researchers to explore theoretical evidence to support medications which targeting AHR signaling pathways.

Here, we mainly summarize the effects of several different virus infections on the AHR signaling pathways, and the effects of the AHR signaling pathway on virus replication and proliferation in turn. We aim to provide a solid theory for the future search for antiviral drugs targeting AHR.

## AHR function

**Fig. 1:**
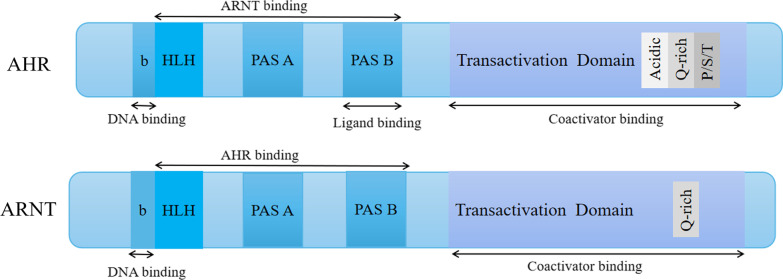
The secondary structure of aryl hydrocarbon receptor (AHR) and aryl hydrocarbon receptor nuclear translocator (ARNT)

AHR is a kind of basic helix–loop–helix (bHLH) Per–Arnt–Sim (PAS) homology domain protein belonging to the bHLH superfamily of transcription factors [10]. The functional domain of AHR protein consists of three parts: the bHLH domain, PAS domain, and a glutamate-rich domain (Fig. 1). The bHLH domain is located at the N-terminus of the AHR protein and assists AHR binding to the promoter region of target genes and protein dimerization. The PAS domain assists in the formation of protein complexes by linking to the AHR nuclear transporter (ARNT) and binding to ligands. The C-terminal region is a glutamate-rich domain that plays a role in recruitment and transcriptional activation [11]. AHR was first discovered as a hydroxylase "inducer" by Poland and Glover in 1973 by using environmental chemicals as probes [12]. In 1974, mice with different genetic backgrounds were found to show different susceptibility to environmental chemical 2,3,7,8-tetrachlorodibenzo(p)dioxin (TCDD), possibly as a result of the polymorphisms in this unidentified hydroxylase activator [13, 14]. With the sequencing of the highly conserved N-terminal sequence of AHR in 1991 [15], and the cloning of the AHR gene in 1992 [16, 17], came a better understanding of the AHR as receptors of carcinogenic environmental ligands. Over time, a variety of environmental chemicals, including PAHs, aromatic amines, and non-ortho-substituted planar polychlorinated biphenyls (e.g., PCB-118, PCB-156, PCB-126), were shown to act largely through the AHR [18]. For example, after being stimulated by these chemical ligands, AHR can be transported from the cytoplasm to the nucleus and bind to another protein, ARNT (Fig. 1), to form a heterodimer. This heterodimer targets downstream target genes, activating the corresponding genes' abnormal expression, such as cytochrome P450 1A1/ cytochrome P450 1B1 (CYP1A1/CYP1B1), ultimately leading to cell toxicity, interference with animal endocrine, immunotoxicity, and even occurrence of cancer [10].

The activation process of AHR involves changes in various protein modifications, such as phosphorylation, ubiquitination, and SUMOylation, which regulate the protein's localization, activity, and stability [19]. Phosphorylation modification mainly affects the localization of AHR in cells. There are 3 phosphorylation motifs of protein kinase C (PKC) on AHR nuclear localization signal (NLS): S12, T22, and S36. Among them, the phosphorylation of S12 and S36 negatively regulates the nuclear entry of AHR and affects the binding ability of AHR to DNA. Phosphorylation of residues near nuclear export signal (NES) regulates AHR localization in cells. Phosphorylation of S68 in AHR NES by mitogen-activated protein kinases 38 (p38) enables AHR to be transported from the cytoplasm to the nucleus. After AHR enters the nucleus, S36 is phosphorylated again, enhancing the AHR complex's activity and promoting gene transcription [20]. The ubiquitination mainly mediates the degradation of AHR after activation, thus playing a regulatory role. The ubiquitination site of AHR is located in the transactivation domain (TAD). Degradation by ubiquitination partially depends on forming the AHR/ARNT heterodimer and binding to DNA [21]. Besides, as mentioned above, AHR can also act as an E3 ubiquitin ligase to promote the ubiquitination and degradation of some sex hormone receptors. SUMOylation enhances AHR stability through inhibition of its ubiquitination. However, this may suppress its transcriptional activity [22].

Besides regulating xenobiotic metabolism, AHR has had many alternative functions since its discovery. Several articles have reported that virus infections can affect the AHR signaling pathways, which play multiple roles in cells. On the one hand, AHR has associated with virus pathogenic response [7]. Specifically, ocular infections caused by the herpes simplex virus (HSV) lead to a chronic immune-inflammatory response leading to blindness when the eye is repeatedly infected. However, a single dose of TCDD in a mouse model alleviates herpetic keratitis lesions, reduces viral load, and reduces pro-inflammatory sex cytokines. However, FICZ, an endogenous AHR ligand, did not exhibit the same efficacy, suggesting that activation with different classes of ligands may have different biological effects and that nontoxic AHR agonists may have the potential capacity to treat HSV-induced eye infections [23].

On the other hand, in some virus-infected cells, the interaction between virus genes and host genes, and virus-encoded products can affect the AHR signaling pathways, affecting the occurrence and development of some diseases. Recently Federico et al. discovered that AHR is the host factor of the Zika virus and a candidate target for antiviral therapy [24]. Zika virus infections will promote the activation of the AHR signaling pathways, and the activation of the AHR signaling pathways will, in turn, trans-activate virus replication, thus resulting in congenital Zika syndrome characterized by fetal brain abnormalities [25] and also Guillain–Barré syndrome (GBS) [26]. All evidence suggests that AHR plays a crucial role in virus infections.

## AHR signaling pathways

Compounds from the exogenous environment or appear as by-products of endogenous metabolism can bind to the AHR to trigger adaptive cellular responses through different signaling pathways [27]. Undoubtedly, in vertebrates, AHR is widely expressed in multiple cell types and is involved in regulating fundamental cellular processes, such as cell proliferation, differentiation, and stress responses. Initially, it was thought that AHR ligands were all exogenous chemicals like TCDD. However, in addition to those exogenous substances, many endogenous and natural compounds have also been identified as effective ligands of AHR [28]. Typical endogenous candidates are a class of metabolic intermediates produced from tryptophan (Trp). Among them, the tryptophan metabolite kynurenine (Kyn), catalyzed by indole 2,3-dioxygenase (IDO) and tryptophan 2,3-dioxygenase (TDO), has been widely studied [29]. IDO1 is the rate-limiting enzyme for kynurenine production and is easily induced by inflammatory cytokines such as IFN-γ.

Ligand-specificity of AHR signaling is multifactorial and influenced by pharmacokinetic aspects, compound-specific conformational changes of AHR and other parameters [30]. Recently, researcheres elucidated the molecular mechanisms underlying the ligand-specific differences in the AHR response of human epithelial cells [31]. The complex interplay between characteristics of the ligand and cell type contributes to the consequences of AHR signaling pathways [32]. Here, we divide it into two categories: genomic and non-genomic pathways.

### AHR genomic pathways

Inactive AHR forms a protein complex with two heat shock protein 90 (Hsp90), hepatitis B Virus X-associated protein 2 (XAP2), co-partner p23, and c-SRC protein kinase in the cytoplasm [33]. HSP90 is involved in the folding of newly synthesized AHR, which can stabilize the conformation of AHR and maintain AHR in an inactive state that can bind to ligands. XAP2 anchors AHR on the cytoskeleton and prevents AHR from being incorporated into nucleoproteins. On the other hand, these molecular chaperones can inhibit the ubiquitination and degradation of AHR, thereby maintaining the amount of AHR in the cytoplasm at a certain level [34]. Once engaged with exogenous or endogenous ligands, the AHR sheds XAP2 and Src and translocates to the nucleus [35]. In the nucleus, the AHR binds with the ARNT and forms an active heterodimer, which modulates the expression of target genes by binding to xenobiotic responsive elements (XRE, 5’-CGCGTG-3'), thus recruiting coactivators including NCoA-2 and p/CIP [36] and transactivating a variety of genres including the hydroxylases CYP1A1 and CYP1B1, which metabolize some environmental AHR ligands into mutagenic epoxide intermediates [37].

**Fig. 2:**
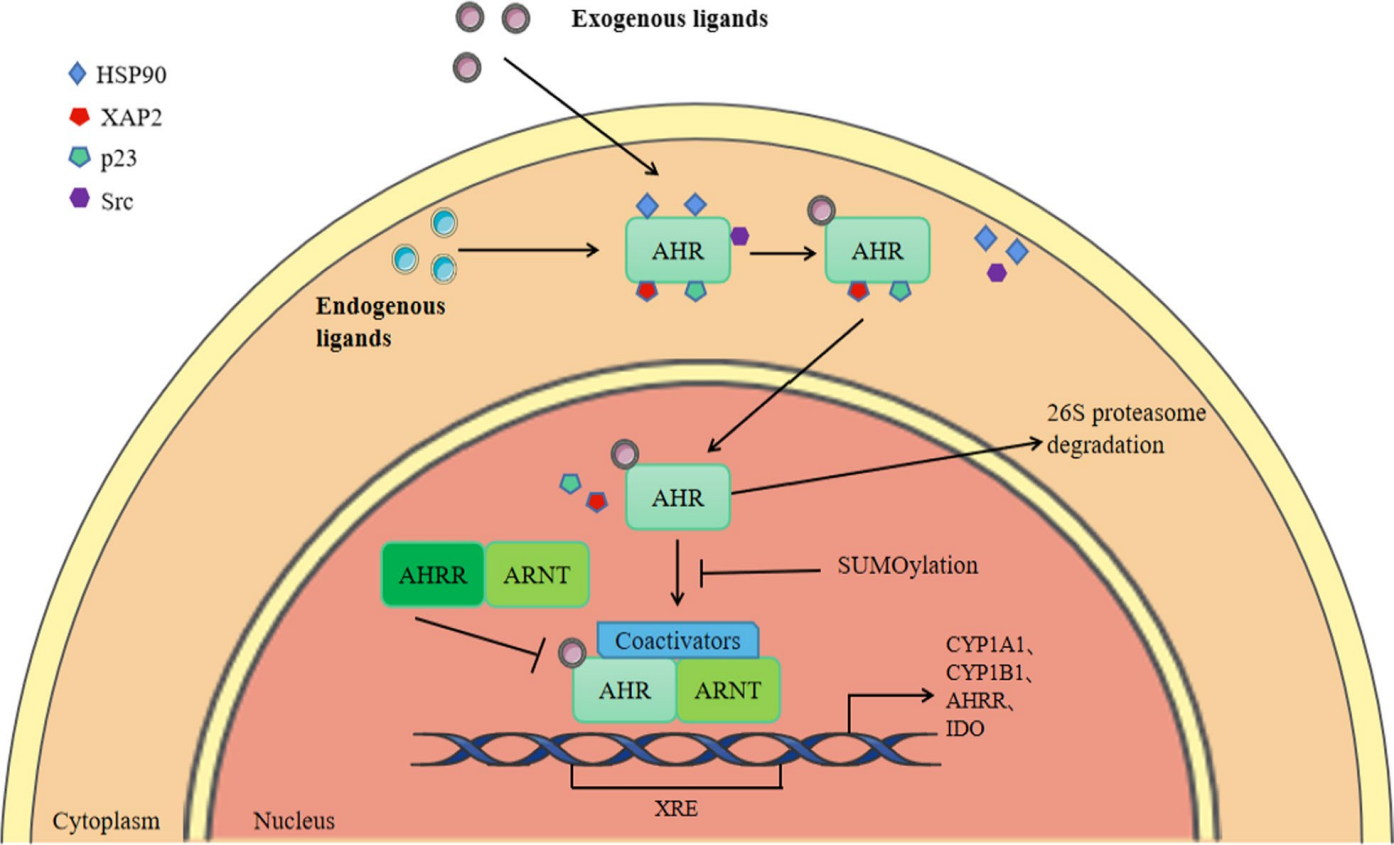
The AHR genomic pathway. Inactive AHR forms a protein complex with two HSP90, XAP2, p23, and SRC in the cytoplasm. Once engaged with exogenous or endogenous ligands, the AHR sheds XAP2 and Src and translocates to the nucleus. In the nucleus, the AHR binds with the ARNT and forms an active heterodimer, which modulates the expression of target genes by binding to xenobiotic responsive elements (XRE, 5ʹ-CGCGTG-3ʹ), thus recruiting coactivators including NCoA-2 and p/CIP and transactivating a variety of genres including the hydroxylases CYP1A1, CYP1B1, AHRR, and IDO

One of the most important target genes activated in the AHR genomic pathway is the AHR repressor (AHRR) (Fig. 1) [38]. The AHRR protein has a similar structure to AHR but cannot bind ligands because of the defect of the PAS B domain in the N-terminal region [39]. Moreover, the AHRR is also different from AHR and ARNT in the C-terminal domain. In AHRR, it is a transrepression domain rather than a transactivation domain in AHR and ARNT. It can recruit corepressors involved in a negative feedback loop for AHR. Under these circumstances, AHRR suppresses AHR activity by binding to ARNT and XRE (AHRR-ARNT complex) (Fig. 2) [40, 41]. In this way, AHRR can regulate the transcription process of AHR-dependent genes. After being exported out of the nucleus, the AHR is rapidly degraded in the cytoplasmic compartment by the 26S proteasome [42].

### AHR non-genomic pathways

**Fig. 3:**
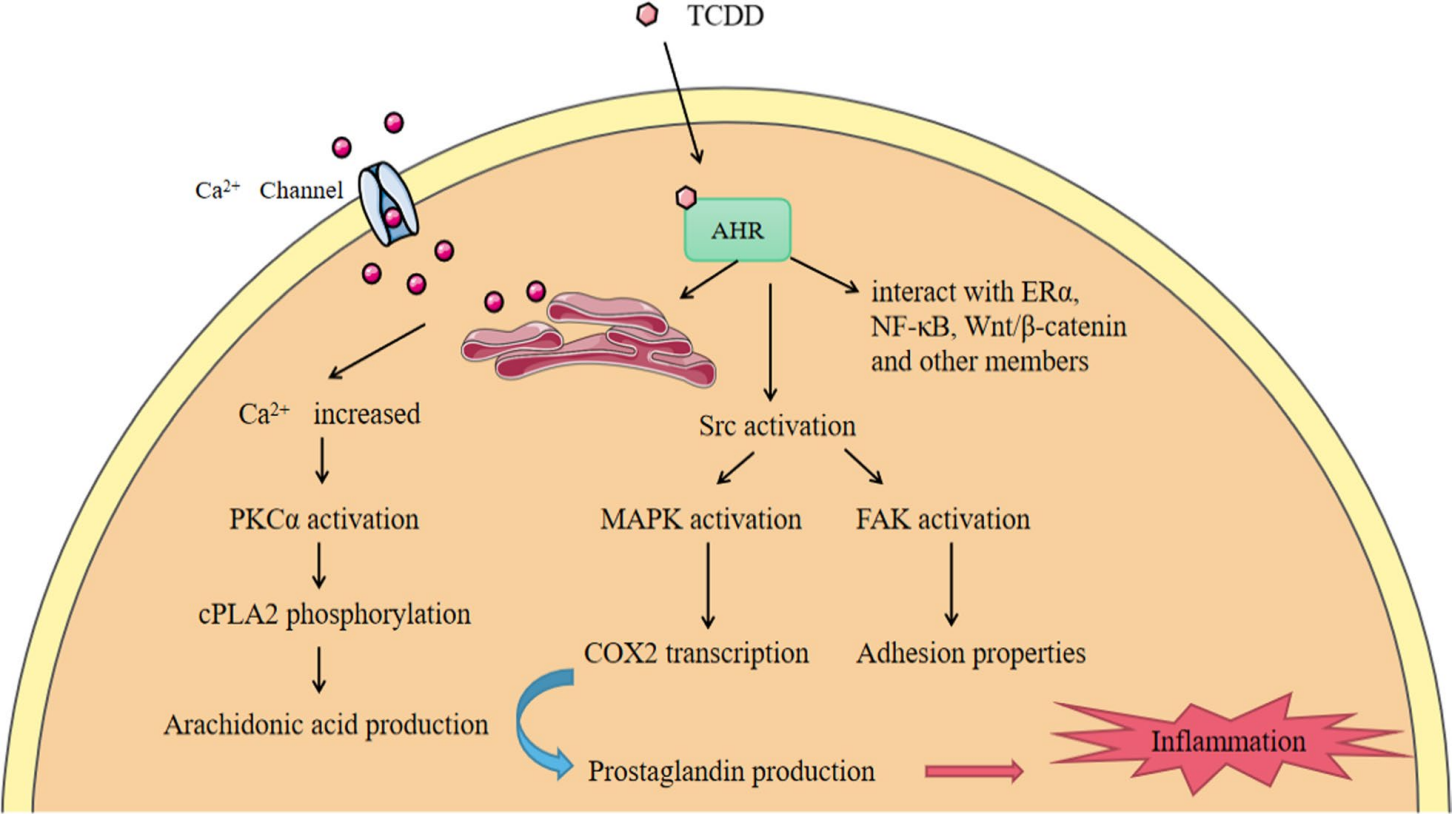
The AHR non-genomic pathways. After exogenous ligand TCDD binds to AHR, calcium ions from the extracellular and endoplasmic reticulum enter the cytoplasm, resulting in increased intracytoplasmic calcium ion concentration rapidly, which causes the activation of PKCα, the phosphorylation of PLA2 and the subsequent production of arachidonic acid. At the same time, the binding of TCDD to the AHR results in the release of tyrosine kinase Src from the AHR complex. The activation of Src could be accompanied by the activation of the FAK and the modification of the adhesion properties. The activation of Src could also directly lead to the rapid activation of MAPK signaling pathways. Next, the activation of MAPK by Src leads to the transcription of COX2, and it can use arachidonic acid to produce prostaglandins which can cause inflammation. At last, these two TCDD-activated signaling pathways ultimately converge toward the stimulation of inflammation

In addition to the genomic pathways, several non-genomic pathways have been found in recent years (Fig. 3). For example, after TCDD binds to AHR, calcium ions from the extracellular and endoplasmic reticulum enter the cytoplasm, resulting in increased intracytoplasmic calcium ion concentration rapidly, which causes the activation of PKCα, the phosphorylation of phospholipase A2 (cPLA2) and the subsequent production of arachidonic acid [43]. At the same time, the binding of TCDD to the AHR results in the release of tyrosine kinase Src from the AHR complex [44]. The activation of Src could be accompanied by the activation of the Focal Adhesion Kinase (FAK) and by the modification of the adhesion properties of the cell through the disruption of focal adhesion points [44, 45]. Furthermore, the activation of MAPK by Src leads to the transcription of cyclooxygenase 2 (COX2), which can promote the production of prostaglandins. At last, these two TCDD-activated signaling pathways ultimately converge toward the stimulation of inflammation [46]. At the same time, the AHR could also play its role by interacting with Wnt/β-catenin [47], ERα [48], or NF-κB [49], and these transcription factors also impact AHR signaling correspondingly. It is not difficult to find an interaction effect between AHR and the above signaling pathways. For example, β-catenin is now described as a co-activator of this receptor [8].

## The effects of virus infections on AHR signaling pathways

Different viruses have different effects on AHR signaling pathways. Here we mainly introduce 7 viruses (Table 1).

**Table 1:** Effects of virus infections on AHR signaling pathways

Types	Virus	Effects of AHR signaling pathways after virus infections	Corresponding influence	Reference
RNA	ZIKV	Promote the synthesis of AHR endogenous ligand Kyn by upregulating IDO1 and TDO2, thereby activating the AHR signaling pathway	Inhibit IFN-I expression and reduce the body's antiviral ability; at the same time, after activation of AHR, it can inhibit the NF-κB signaling pathway to limit the intrinsic immunity driven by PML protein, which is conducive to the replication of ZIKV in vivo	[24]
	SARS-CoV-2	Promote the synthesis of AHR endogenous ligand Kyn via promoting the expression of IFN-β、γ to upregulate IDO1, thereby activating the AHR signaling pathway	Stimulate mucins production, thereby promoting hypoxia by hindering O_2_ diffusion at alveolar sites; on the other hand, AHR activation, in turn, promotes virus replication	[57] [59]
	HCV	Activate the AHR signaling pathway by upregulating the AHR endogenous ligand Kyn	After activation of AHR, the expression of its downstream target gene CYP1A1 increases, promoting the formation of LDs and accumulation of liver fat, which is closely related to the progression of HCC	[62]
	IAV	Possibly activate the AHR signaling pathway by upregulating Kyn levels	Diminish host responses and reduce cytotoxic T lymphocytes production, thereby reducing cellular antiviral immunity	[102]
	HTLV-1	AHR is activated, but the exact mechanism is unknown	Facilitate HTLV-1 plus-strand transcription and control viral latency-reactivation switching	[104]
	HIV-1	In the lymphocyte, AHR activation promotes HIV-1 infection and reactivation; in the macrophage, AHR activation causes a block to HIV-1 replication	In the lymphocyte, AHR directly binds to the HIV-1 5′-LTR; in the macrophage, AHR activation surpresses the transcription of CDK1, CDK2, resulting in dNTP depletion and antiviral effects	[70, 71]
DNA	EBV	EBV-encoded EBNA3 facilitates the role of the AHR pathway by promoting AHR nuclear translocation in B lymphocytes; EBV-encoded LMP1 affects AHR signaling pathway by activating NF-κB in B lymphocytes; EBV-encoded LMP2A suppresses the role of the AHR pathway through the ERK signal pathway in EBV-associated gastric cancer	In B lymphocytes, EBNA3 activates AHR, which in turn promotes EBV to enter the lytic phase, and it is conducive to virus replication; In gastric cancer cells, inhibition of AHR by LMP2A favors EBV to maintain a latent state	[72] [78] [93]
	HCMV	Increase Kyn levels by promoting Kyn synthesis and inhibiting Kyn metabolism in fibroblasts, thereby activating the AHR signaling pathway	AHR activation following HCMV infection, in turn, promotes efficient production of viral progeny; And it promotes HCMV-Induced G1/S block to cell cycle progression, thereby preventing cell proliferation and preserving metabolic resources for viral progeny	[100]

### Zika virus

Zika virus (ZIKV) is an enveloped, single positive-stranded RNA virus in the Flavivirus genus of the Flaviviridae family and is associated with various congenital disabilities, including microcephaly, known as congenital ZIKV syndrome [50]. Recently, Giovannoni et al. (2020) observed that the AHR signaling pathway could be activated by ZIKV infection [24]. Initially, the researchers found that ZIKV infection significantly impacts the AHR signaling pathway by RNA sequencing of ZIKA-infected human hepatocytes HepG2. After ZIKV infection, the expression of AHR downstream target genes CYP1A1 and CYP1B1 increased. Through further exploration, it was identified that ZIKV could upregulate the activity of endogenous AHR ligand Kyn by increasing the activities of the enzymes IDO1 and TDO2 that synthesize Kyn, thereby activating the AHR signaling pathway. The authors also confirmed that the up-regulation of AHR can drive immune regulatory mechanisms to achieve immune escape, thereby promoting ZIKV replication and triggering a series of virus infection-related diseases, such as congenital ZIKV syndrome. Furthermore, through animal experiments, the researchers thoroughly verified that two types of AHR antagonists could effectively improve the related symptoms induced by ZIKV infection and identified AHR as a host factor of ZIKV infection and a candidate target for antiviral therapy.

### Severe acute respiratory syndrome coronavirus 2 (SARS-CoV-2)

SARS-CoV-2 is an enveloped single positive-stranded RNA virus of the family Coronaviridae [51] and is the causative agent for coronavirus disease 2019 (COVID-19) [52]. Due to the continuous mutation of the virus, although we have developed different types of vaccines against it [53], the spread of this virus has not completely stopped within a certain range until today. COVID-19 initially presents with "flu-like" symptoms and mainly manifests as lung damage but may also affect other organs [54, 55]. Once COVID-19 enters a severe stage, it becomes very difficult to manage and causes patient death [56]. So it is significant to elucidate the underlying mechanism of the pathogenicity of this virus.

In 2020, it was found that after SARS-CoV-2 infection, mucins expression can be enhanced by activating the IFN-AHR signaling pathway, thereby inducing hypoxia in patients [57]. Specifically, during SARS-CoV-2 infection, the body will increase the expression of IFN-β and IFN-γ to fight the virus. After the increase of IFNs, it can upregulate the enzyme IDO1 that synthesizes Kyn, thereby promoting the activation of the AHR signaling pathway, resulting in increased mucins expression in alveolar epithelial cells. Excess mucins adhere to the blood-gas barrier and increase its thickness. The thickened barrier gradually impedes the normal gas exchange of O_2_ and CO_2_, inducing hypoxia in the patient [58]. Briefly, by conducting experiments at the cellular, tissue, and animal levels, the researchers verified that targeting the IFN-AHR signaling pathway may be a potential strategy for effective treatment of patients with COVID-19 and provided a new therapeutic idea for dyspnea caused by SARS-CoV-2 infection.

Another research also reported that different types of coronaviruses could activate the AHR signaling pathway, not only SARS-CoV-2 but also Middle East respiratory syndrome coronavirus (MERS-CoV), human coronavirus 229E (HCoV-229E), SARS-CoV-1 and murine coronavirus (M-CoV) [59]. In addition, the team found a correlation between AHR expression and viral load in SARS-CoV-2 infected patients. The activation of AHR could, in turn, promote coronavirus replication. After treatment with AHR antagonists in vitro, it was found that the replication of HCoV-229E, the pathogen of the common cold, and SARS-CoV-2, the pathogen of COVID-19, was inhibited to some extent. Altogether, these findings suggest that AHR activation is a common strategy for coronaviruses to evade host immune responses and promote self-replication and that AHR activation may further promote lung-related pathological changes.

### Hepatitis C virus

As is known to all, the Hepatitis C virus (HCV) is a flavivirus belonging to the Hepacivirus genus. HCV infection can result in persistent diseases, keeping asymptomatic for years before developing severe liver pathology, including cirrhosis and hepatocellular carcinoma (HCC) [60]. After HCV infection, host cell metabolism is altered, producing specialized membrane structures and altering organelles, such as double-membrane vesicles and enlarged lipid droplets (LDs), which enable virus replication and assembly [61]. However, the molecular mechanisms of HCV-host interaction are largely unknown. Recently, some researchers found that HCV infection can activate the AHR signaling pathway and upregulate the AHR downstream target gene CYP1A1, promoting the production of LDs. Subsequently, the accumulated LDs can promote the efficient production of progeny viruses [62].

We hypothesize that AHR can regulate the production of triglycerides and LDs, which to some extent determine the replication ability of HCV in host cells. It has also been demonstrated that the transcriptional activity of AHR is elevated in HCV-infected cells, and the level of the AHR endogenous ligand Kyn is also elevated in HCV-infected patients [63]. In addition, lipid accumulation in the liver may also be the basis for the development of HCC, and the lipid accumulation caused by the AHR-CYP1A1 pathway may be closely related to the development of HCC [64].

As early as 2016, Canavese et al. proposed a hypothesis based on the current experimental progress and evidence: After HCV infection, cells promote HCC by regulating the TDO-Kyn-AHR signaling pathway, leading to tumorigenesis. They believe that changes in the expression of AHR pathway-specific genes are associated with the progression of HCV infection and HCC. Interestingly, some researchers recently found that aflatoxin B1 (AFB1), closely related to HCC, can play a role similar to AHR ligand, promote AHR nuclear translocation, and activate the AHR signaling pathway [65].

### Human immunodeficiency virus type-1 (HIV-1)

Human immunodeficiency virus type-1 (HIV-1) is an important infectious agent that is responsible for acquired immunodeficiency syndrome (AIDS) [66]. As a kind of successful retrovirus, HIV-1 remains a global health problem of unprecedented dimensions [67]. More than a decade ago, researchers found that AHR activation stimulated by ligand of TCDD or by TCDD chemical homologue 3-methylcholanthrene 3-MC was shown to reactivate HIV-1 from latency [68, 69]. In recent years, through further exploration, researchers demonstrated that AHR was activated by Trp metabolites to promote HIV-1 infection and reactivation [70]. Mechanically, they confirmed that AHR directly binds to the HIV-1 5′ long terminal repeat (5′-LTR) at the molecular level to activate viral transcription and infection, and AHR activation by Trp metabolites increases its nuclear translocation and association with the HIV 5′-LTR. Moreover, they also found AHR could bind to HIV-1 Tat to facilitate the recruitment of positive transcription factors to viral promoters. These findings all suggest that a downstream target AHR may be a potential target for modulating HIV-1 infection.

Another researchers elucidated that the activation of AHR could not always facilitate HIV-1 replication. Tonya et al. showed that AHR activation in macrophages caused a block to HIV-1 replication [71]. To be specific, the activation of AHR downregulates the transcription of cyclin-dependent kinase CDK1, CDK2 and associated cyclins, resulting in dNTP depletion and antiviral effects. Totally, the effect of AHR activation on HIV-1 may be disparate in different cell types. It is still essential for us to further elucidate the effect of AHR signaling pathways on HIV-1 latent state and replication.

### Epstein–Barr virus

Epstein–Barr virus (EBV) was the first definitive human tumor virus as a member of the human gamma-herpesvirus subfamily. EBV is generally latently infected in host cells and encodes corresponding viral products, such as viral proteins and microRNAs. Studies have found that these viral encoded products may affect the AHR signaling pathway, thereby affecting the occurrence and development of tumors. Here we mainly introduce the effects of three EBV-encoded protein products on the AHR pathway.

#### EBNA3 facilitates the role of the AHR pathway by promoting AHR nuclear translocation in B lymphocytes

EBV nuclear antigen 3 (EBNA3) is one of the EBV-encoded nuclear antigens indispensable for immunoblastic transformation and sustains the proliferation of B lymphocytes [72]. When EBV infects and maintains latency in B lymphocytes, its encoded viral product protein EBNA3 can interact with XAP2 and AHR. They influence the localization of each other in cells. When exogenous ligands, such as TCDD, interact with the AHR complex, the cytoplasmic localization of AHR affected by XAP2 is counteracted by EBNA3, resulting in nuclear translocation of AHR, which enhances the AHR signaling pathway. It has now been demonstrated that the stability of EBNA3 interaction with AHR is determined by the activation state and the presence of XAP2. Meanwhile, the interaction of EBNA3 with Hsp90 is mediated through XAP2 [73]. It is noteworthy that the nuclear translocation effect of EBNA3 on the AHR is only functional when TCDD acts as a ligand. Without TCDD, the cytoplasmic localization of AHR by XAP2 would be dominant and stronger than the nuclear translocation of AHR by EBNA3. Under this circumstance, EBNA3 cannot promote the nuclear translocation of AHR.

**Fig. 4:**
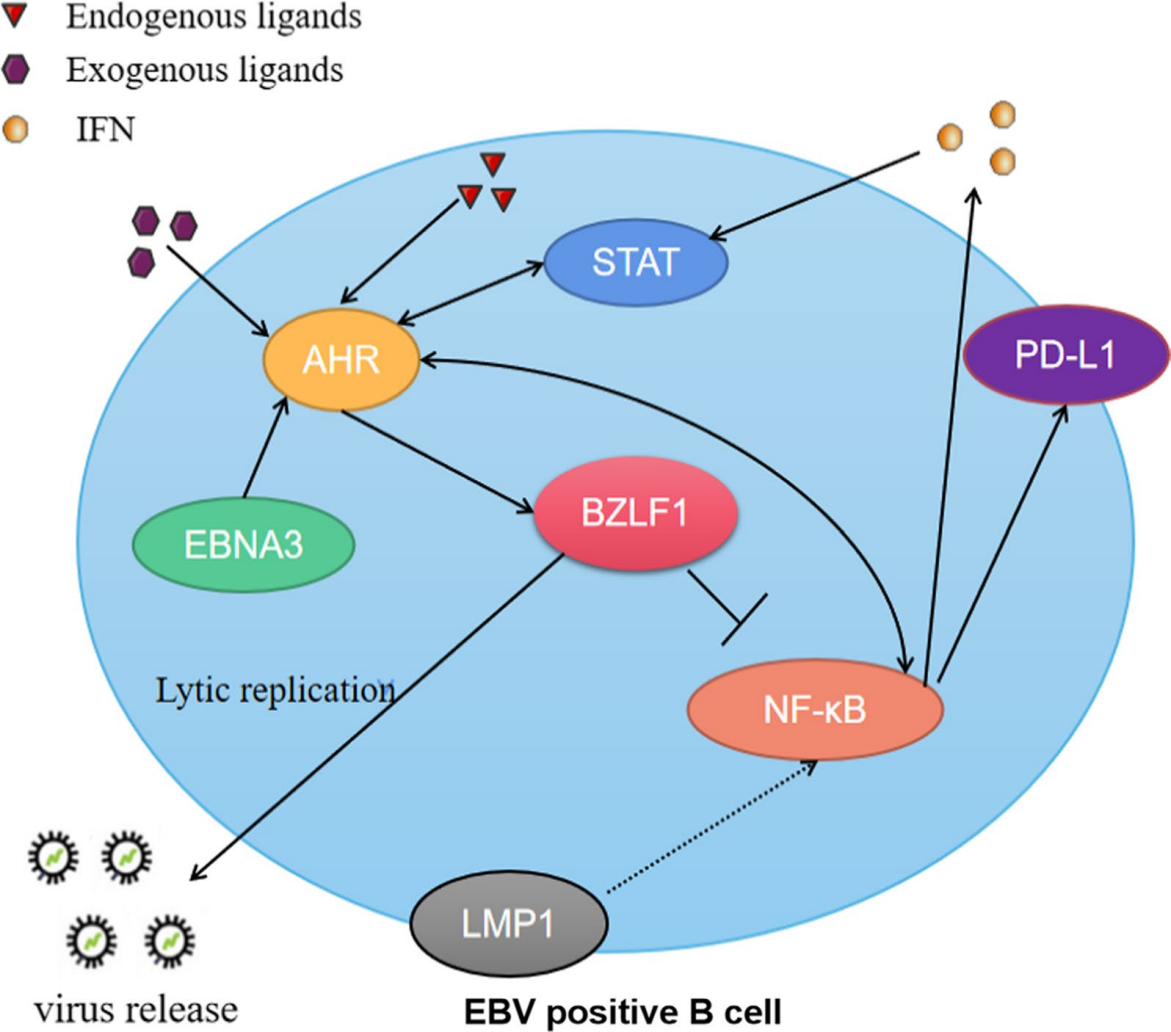
Effects of Epstein-Barr virus-encoded products on AHR signaling pathway in B cells. Distinct endogenous and exogenous AHR ligands regulate the metabolism of B cells. EBV-encoded protein EBNA3 enhances dioxin-induced AHR transcriptional activity, which induces the expression of BZLF1. BZLF1 promotes the virus to enter a lytic replication state, inhibits the NF-κB signaling pathway, and affects the expression of PD-L1. EBV-encoded LMP1 can affect the AHR signaling pathway by activating NF-κB

The facilitation effect of EBNA3 on the AHR signaling pathway activated by TCDD was confirmed by Elena V. Kashuba et al. in 2005 [73]. Following this, it was found that the AHR signaling pathway promoted by EBNA3 was associated with EBV reactivation. It has been demonstrated that the AHR complex and EBNA 3 reactivate EBV's immediate-early viral transactivator, BZLF1 [74]. This initiating factor of lytic replication counteracts latent viral signals to a certain extent by blocking the NF-κB signaling pathway (Fig. 4) [75].

**Fig. 5:**
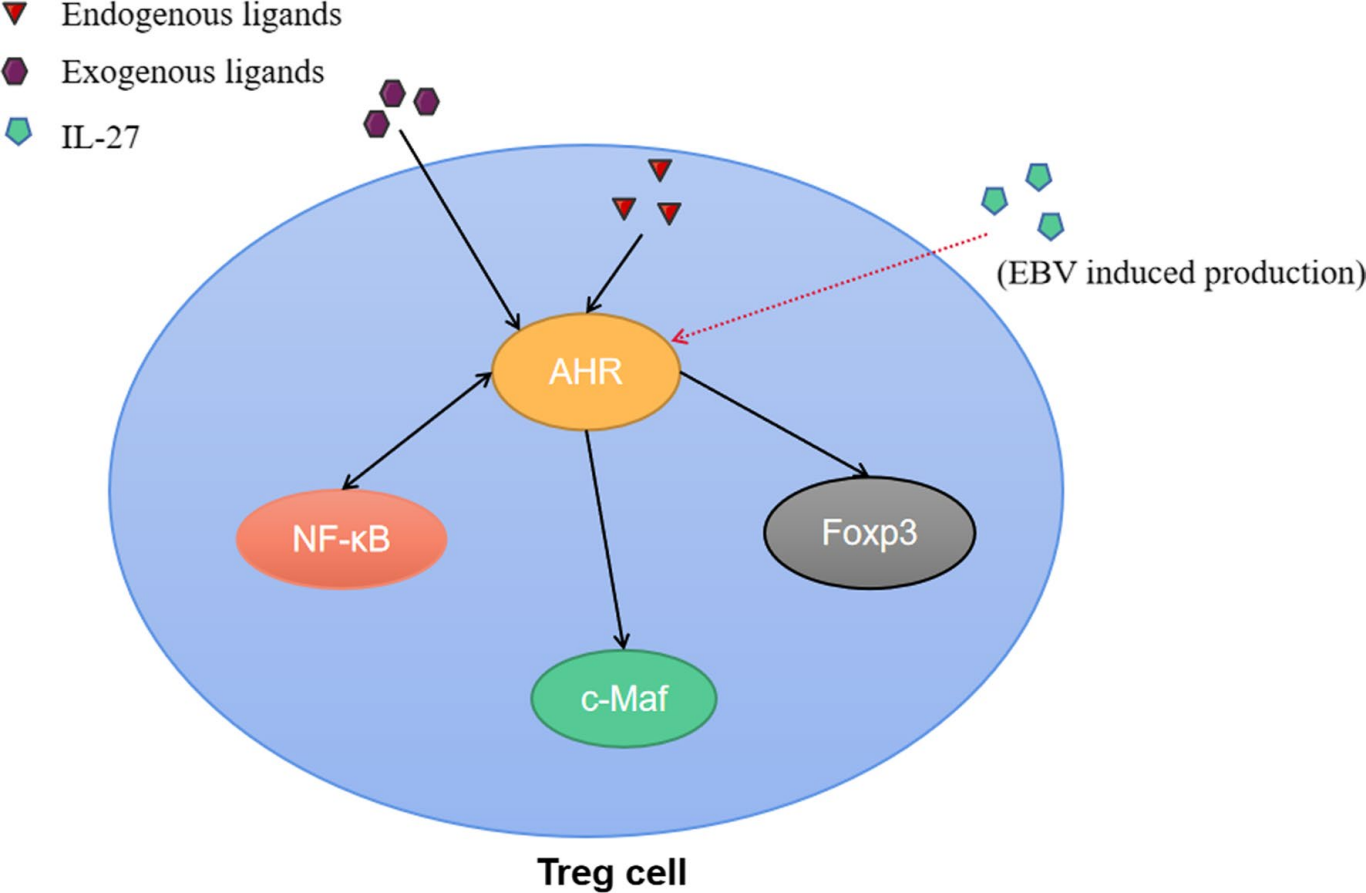
Effects of Epstein-Barr virus-encoded products on AHR signaling pathway in Treg cells. EBV-induced production of IL-27 enhances AHR expression, promoting transcriptional activity of c-Maf and Foxp3. There is also an interaction between the AHR signaling pathway and the NF-κB signaling pathway

The expression of AHR in T cells is also induced by the cytokine IL-27. In these cells, AHR interacts with c-Maf, a transcription factor identified in a subset of Tregs [74, 75]. These CD4^+^ CD25^+^ Foxp3^−^ c-Maf^+^ Treg cells, termed Treg-of-B cells, are formed in response to repeated interactions with B cells [76]. Treg-of-B cells express PD-1 and additional checkpoints in regulating Th2, Th1, and Th17 responses under physiological cues that have yet to be fully elucidated (Fig. 5).

#### LMP1 affects AHR signaling pathway by activating NF-κB in B lymphocytes

EBV is an established factor in systemic lupus erythematosus (SLE) and PD-1 immunobiology [77]. The role of the PD-1 receptor and its ligands in the disease progression of SLE has been identified. In recent years, it has been found that EBV-encoded products can regulate the expression of PD-1 and 2 by affecting the AHR signaling pathway and other pathways that interact with them, such as NF-κB and/or STAT1, thereby affecting the development of SLE (Fig. 5) [78]. The EBV-encoded product latent membrane protein 1 (LMP1) can induce the activation of NF-κB and produce various cytokines in B cells, such as IL-27 subunit, EBI3, BAFF, APRIL, IFN-α, IFN-γ [79, 80]. The newly generated IFN subsequently induces STAT1 activity [81], and these signals, as mentioned earlier, may ultimately play a role in inducing PD-L1 expression in EBV latently infected cells [82]. If more PD-L1 is expressed on B cells, then more PD-1 is attached to it on Th cells, suggesting more severe SLE [83, 84]. Since LMP1 activates the NF-κB pathway to some extent, and there is an interaction between the NF-κB pathway and the AHR signaling pathway[85], we speculate that LMP1 may indirectly affect the AHR signaling pathway by regulating the NF-κB pathway.

#### LMP2A suppresses the role of the AHR signaling pathway through the ERK signaling pathway in EBV-associated gastric cancer

It was reported that AHR was highly expressed in many types of human malignant tumor tissues and cell lines and was involved in the occurrence of tumors [86, 87]. Medical-related statistics have proved that compared with precancerous lesions, the expression of AHR in gastric cancer (GC) tissue is significantly increased [88]. Latent membrane protein 2A (LMP2A) is one of the EBV-encoded products and is expressed in more than 50% of EBV-infected GC cases [89]. LMP2A not only plays a key role in maintaining the latent state of the virus but also participates in the regulation of various intracellular signaling pathways, such as MAPK/ERK, phosphoinositide 3-kinase/protein kinase B (PI3K/AKT) and NF-κB pathway, and is an important molecule in the carcinogenic process [90]. Studies have shown that LMP2A regulates the expression of certain genes by modulating these pathways, affecting tumor progression [91, 92]. Therefore, in recent years, researchers have studied the activation of the AHR pathway in EBV-associated gastric cancer (EBVaGC) and EBV-negative gastric cancer (EBVnGC) cell lines to observe the effect of EBV infection on the AHR pathway in GC cells [93].

Early exploration has proved that LMP2A can activate the MAPK/ERK pathway and promote the accumulation of p-ERK in gastric cancer cells. It also provided direct evidence that the inhibition of AHR expression by LMP2A may be achieved by activating the ERK pathway[93]. There are still many controversies about the role of AHR in tumors. With further study, the researchers found that EBV-infected cells were less sensitive to AHR agonists than EBVnGC cells [92]. Although it limited the progression of cancer on the one hand, which may be related to EBVaGC low lymphatic metastasis and good prognosis, it may also increase the difficulty of treatment with AHR as the target on the other hand. Furthermore, the finding that LMP2A suppresses the role of the AHR pathway through the ERK signal pathway in EBVaGC may provide an important direction for the future treatment of EBVaGC, and we still need more exploration about the effects of EBV infection on AHR signaling pathway.

### Human cytomegalovirus (HCMV)

HCMV is a beta-herpesvirus that establishes lifelong asymptomatic infection in most people and is one of the leading causes of congenital disability [94, 95]. After the virus infects cells, it manipulates various aspects of the metabolism to facilitate its replication and spread [96, 97].

Previous studies have found that ectopic expression of the HCMV IE1 protein induces the accumulation of IDO1 RNA levels [98] and reduces the accumulation of kynureninase RNA in fibroblasts [99], which is the enzyme that synthesizes Kyn and catabolizes Kyn, respectively. Therefore, IE1 can enhance the level of Kyn by promoting the synthesis of Kyn and/or reducing the consumption of Kyn. In recent years, some scholars have verified that HCMV infection can activate AHR by upregulating Kyn and activating AHR requires viral gene expression. At the same time, AHR contributes to the efficient production of HCMV progeny. After AHR is activated, it can broadly affect the transcriptome of infected cells. In addition, the authors also found that AHR can promote HCMV-induced G1/S block to cell cycle progression, thereby preventing cell proliferation and preserving metabolic resources for viral progeny [100].

Paradoxically, another research team observed that the expression of hypoxia-inducible factor 1α (HIF1α) was increased after HCMV infection. While exploring the role of HIF in HCMV replication, they found that HIF1α inhibited the concentration of intracellular and extracellular Kyn and the expression of IDO1. HIF1α inhibits AHR activation by regulating the synthesis of the AHR endogenous ligand Kyn, thereby limiting virus replication [101].

The regulation of the AHR signaling pathway after HCMV infection is complex and variable, and it is difficult to clarify its specific and fixed regulatory mechanism, but we can conclude that HCMV infection can indeed exert various effects by affecting the AHR signaling pathway, whether it could prevent cell proliferation or inhibit virus replication.

### Other viruses

The effects of other virus infections on the AHR signaling pathway have also been reported. During primary influenza A virus (IAV) infection, the AHR signaling pathway is activated in immune cells, which in turn inhibits dendritic cells (DC) function and initiates the ability of naive CD8+ T cells to diminish host responses and thereby reduce cytotoxic T lymphocytes production. And the authors identified that AHR activation reduced CD209a expression in DC and CCL17 production in the lung and mediastinal lymph nodes (MLN) during IAV infection [102]. It is well acknowledged that human T-cell leukemia virus type 1 (HTLV-1) establishes latent infection in vivo and can be reactivated under certain circumstances [103]. It has been reported in another article that AHR can play its role as a tunable knob that controls HTLV-1 latency-reactivation switching [104]. Specifically, activated AHR binds to HTLV-1 LTR and drives HTLV-1 plus-strand transcription. It has been demonstrated that HTLV-1 latency-reactivation-latency switching in MT-1 cells can be manipulated by adding and removing additional AHR ligands, suggesting that AHR is a potential target for prophylaxis and treatment of HTLV-1-related diseases.

## Conclusions

In general, our review discusses the relationship between virus infections and AHR pathways, providing an important direction for the future treatment of virus-associated diseases. The activation of the AHR pathway is significantly related to cell proliferation and migration, which also provides the possibility for AHR as a drug target for disease treatment. It is not difficult to see from the above that AHR activation is a common strategy for most viruses to evade anti-virus immunity and promote virus replication. Although the underlying mechanisms by which viruses activate or inhibit AHR vary, their roles are clear: either to evade the host immune responses or promote self-replication and survival. Exploring the impact of different virus infections on the AHR pathway will help us understand the pathogenic mechanism of viruses regulating AHR and provide more precise, efficient, and potential therapeutic targets for antiviral therapy points.

## Data Availability

Not applicable.

## Abbreviations

AHR: Aryl hydrocarbon receptor
DALYs: Disability-adjusted life years
PAHs: Polycyclic aromatic hydrocarbons
HAHs: Halogenated aromatic hydrocarbons
AF: Aminoflavone
SahRMs: Selective AHR modulators
bHLH: Basic helix–loop–helix
PAS: Per–Arnt–Sim
ARNT: AHR nuclear transporter
TCDD: 2,3,7,8-Tetrachlorodibenzo(p)dioxin
PCB: Polychlorinated biphenyl
CYP1A1: Cytochrome P450 1A1
CYP1B1: Cytochrome P450 1B1
AHRR: AHR repressor
ER: Estrogen receptor
AR: Androgen receptor
3-MC: 3-Methylcholanthracene
DDB1: Damaged DNA-binding protein 1
TBL3: Transducin-β-like protein 3
CUL4B: Cullin ubiquitin ligase 4B
Rbx1: RING box protein 1
PKC: Protein kinase C
NLS: Nuclear localization signal
NES: Nuclear export signal
p38: Protein kinases 38
TAD: Transactivation domain
HSV: Herpes simplex virus
GBS: Guillain–Barré syndrome
Trp: Tryptophan
Kyn: Kynurenine
IDO: Indole 2,3-dioxygenase
TDO: Tryptophan 2,3-dioxygenase
Hsp90: Heat shock protein 90
XAP2: Hepatitis B Virus X-associated protein 2
XRE: Xenobiotic responsive elements
cPLA2: Phosphorylation of phospholipase A2
FAK: Focal Adhesion Kinase
MAPK: Mitogen-activated protein kinase
ERK: Extracellular signal-regulated kinase
JNK: Jun N-terminal kinase
COX2: Cyclooxygenase 2
E2: Estrogen 2
ZIKV: Zika virus
SARS-CoV-2: Severe acute respiratory syndrome coronavirus 2
COVID-19: Coronavirus disease 2019
MERS-CoV: Middle East respiratory syndrome coronavirus
HCoV-229E: Human coronavirus 229E
M-CoV: Murine coronavirus
HCV: Hepatitis C virus
HCC: Hepatocellular carcinoma
LDs: Lipid droplets
AFB1: Aflatoxin B1
HIV-1: Human immunodeficiency virus type-1
AIDS: Acquired immunodeficiency syndrome
5′-LTR: 5′ Long terminal repeat
EBV: Epstein-Barr virus
EBNA3: EBV nuclear antigen 3
BZLF1: EBV immediate early viral transactivator
SLE: Systemic lupus erythematosus
LMP1: Latent membrane protein 1
GC: Gastric cancer
LMP2A: Latent membrane protein 2A
EBVaGC: EBV-associated gastric cancer
EBVnGC: EBV-negative gastric cancer
HCMV: Human cytomegalovirus
HIF1α: Hypoxia-inducible factor 1α
IAV: Influenza A virus
DC: Dendritic cells
MLN: Mediastinal lymph nodes
HTLV-1: Human T-cell leukemia virus type 1
